# ICAM-Reg: Interpretable Classification and Regression With Feature Attribution for Mapping Neurological Phenotypes in Individual Scans

**DOI:** 10.1109/TMI.2022.3221890

**Published:** 2023-04-03

**Authors:** Cher Bass, Mariana da Silva, Carole Sudre, Logan Z. J. Williams, Helena S. Sousa, Petru-Daniel Tudosiu, Fidel Alfaro-Almagro, Sean P. Fitzgibbon, Matthew F. Glasser, Stephen M. Smith, Emma C. Robinson, Alzheimer’s Disease Neuroimaging Initiative

**Affiliations:** School of Biomedical Engineering and Imaging Sciences, King’s College London, WC2R 2LS London, U.K., and also with Panakeia Technologies, WC2B 4BG London, U.K.; School of Biomedical Engineering and Imaging Sciences, King’s College London, WC2R 2LS London, U.K.; School of Biomedical Engineering and Imaging Sciences, King’s College London, WC2R 2LS London, U.K., and also with the MRC Unit for Lifelong Health and Ageing, University College London, WC1E 6BT London, U.K.; School of Biomedical Engineering and Imaging Sciences, King’s College London, WC2R 2LS London, U.K.; School of Biomedical Engineering and Imaging Sciences, King’s College London, WC2R 2LS London, U.K.; School of Biomedical Engineering and Imaging Sciences, King’s College London, WC2R 2LS London, U.K.; Department of Clinical Neurology, Oxford Centre for Functional MRI of the Brain (FMRIB), OX3 9DU Oxford, U.K.; Wellcome Centre for Integrative Neuroimaging, University of Oxford, OX1 2JD Oxford, U.K.; Departments of Radiology and Neuroscience, Washington University in St. Louis, St. Louis, MO 63130 USA; Department of Clinical Neurology, Oxford Centre for Functional MRI of the Brain (FMRIB), OX3 9DU Oxford, U.K.; School of Biomedical Engineering and Imaging Sciences, King’s College London, WC2R 2LS London, U.K.

**Keywords:** Brain imaging, deep generative models, feature attribution, image-to-image translation

## Abstract

An important goal of medical imaging is to be able to precisely detect patterns of disease specific to individual scans; however, this is challenged in brain imaging by the degree of heterogeneity of shape and appearance. Traditional methods, based on image registration, historically fail to detect variable features of disease, as they utilise population-based analyses, suited primarily to studying group-average effects. In this paper we therefore take advantage of recent developments in generative deep learning to develop a method for simultaneous classification, or regression, and feature attribution (FA). Specifically, we explore the use of a VAE-GAN (variational autoencoder - general adversarial network) for translation called ICAM, to explicitly disentangle class relevant features, from background confounds, for improved interpretability and regression of neurological phenotypes. We validate our method on the tasks of Mini-Mental State Examination (MMSE) cognitive test score prediction for the Alzheimer’s Disease Neuroimaging Initiative (ADNI) cohort, as well as brain age prediction, for both neurodevelopment and neurodegeneration, using the developing Human Connectome Project (dHCP) and UK Biobank datasets. We show that the generated FA maps can be used to explain outlier predictions and demonstrate that the inclusion of a regression module improves the disentanglement of the latent space. Our code is freely available on GitHub https://github.com/CherBass/ICAM.

## I. Introduction

Brain images represent a significant resource in the development of mechanistic models of behaviour and neurological/psychiatric disease as, in principle, they capture measurable neuroanatomical traits that are heritable, present in unaffected siblings and detectable prior to disease onset [1]. For many complex disorders, however, these features of disease [2], [3] are subtle, variable and obscured by a back-drop of significant natural variation in brain shape and appearance [4], [5]; this makes them extremely difficult to detect.

Traditional approaches for analysis of brain magnetic resonance imaging (MRI) rely on group-wise comparisons between disease and control groups, whereby they compare all images in a global average space through performing image registration to a template. Voxel-based morphometry (VBM) is one such common method [6], which has been used in countless studies of development, ageing and dementia [7], [8], [9], [10], [11]. Other techniques include traditional machine learning analysis based on comparisons of hand-engineered features, for example metrics derived from cortical regions [12], [13], [14], [15], [16], [17], [18], or lesion symptom mapping techniques [19]. More recent methods use Gaussian processes [20] to detect diseased brain tissue as outliers against a normative model, fit at each voxel. While these methods have significantly improved understanding of population average patterns of disease [7], they rely on spatial normalisation and therefore lose power at the cortex due to the impact of cortical heterogeneity [4], [21]. This also means that they are not tuned to detect features of disease specific to the individual, which are extremely important for diagnosis and prognosis.

To address these limitations, recent studies have started to apply deep learning methods to brain imaging datasets. Deep learning is state-of-the-art for many image processing tasks [22], and has shown strong promise for brain imaging applications such as healthy tissue and lesion segmentation [23], [24], [25], [26]. Importantly, by design it can work independently of any requirement for spatial normalisation. However, deep learning methods do not, by default, return explanations of the reasoning behind their predictions, leading to them traditionally being referred to as “black box” models.

More recently, several approaches have been developed to make these networks more interpretable through identifying class-relevant features for a particular input. These include post-hoc saliency based methods, designed to detect which features of a specific image contribute most strongly to a class prediction. These typically analyse the gradients or activations of the network, with respect to a given input image, and include approaches such as Gradient-weighted Class Activation Mapping (Grad-CAM) [27], SHAP [28], DeepTaylor [29], integrated gradients [30], guided backpropagation (backprop) [31], and Layer-wise backpropagation (LRP) [32]. In addition, perturbation methods such as occlusion [33] change or remove parts of the input image to generate heatmaps, by evaluating its effect on the prediction.

Such methods have now been applied in various medical imaging applications including in MRI and Positron Emission Tomography (PET) imaging datasets for Alzheimer’s (AD) [34], [35], [36], [37] and Multiple Sclerosis (MS) [38] classification, and cancer detection through breast density regression [39]. However, while in principle, these methods can be applied to detect features from individual images, the results are typically low resolution and noisy, which makes them hard to interpret. Often this leads to studies estimating a group average to aggregate results across individuals, and boost signal to noise to make stable population-wide inferences [36], [37]. This loses individual specificity, and since these feature attribution (FA) methods often detect similar features in both healthy and disease groups, it is difficult to interpret the results.

In addition, since these FA methods are applied to a CNN following training, their power is limited by the constraints of the network they are applied to. Such networks need only focus on the most consistent or discriminative features, sufficient to accurately predict each class. This is a particular issue for medical imaging where diagnosis and treatment rely on comprehensive capture of all features of disease [34], [35], [36], [37], [38], [40]. For example, when applying LRP and guided backprop to brain MRI, it was found that while they were able to detect homogeneous brain structures such as the hippocampus, they were unable to detect heterogeneous structures such as cortical folds [36], [37].

For these reasons, new approaches have recently been proposed which seek holistic explanations for a phenotype through learning to translate images from one class to another [40], [41], [42], [43], [44], [45]. For example Lenis et al. [43] identifies salient regions of any input image by identifying the smallest feasible perturbation that would change a predictor’s score. Similarly, Schutte et al. [44] trains a StyleGAN [46] to simulate osteoarthritis in knee X-ray images and [47] modifies a CycleGAN [45] to generate the minimum pertubation required to change the disease class of retinal images. Most similar to this work is Baumgartner et al. [40], which uses a visual attribution (VA) GAN to translate images classed as Alzheimer’s (AD) to instead resemble Mild Cognitive Impairment (MCI). However, while this method was able to detect more features of disease relative to post-hoc methods, it was still unable to identify much of the phenotypically variable changes around the cortex [48].

**Fig. 1.**
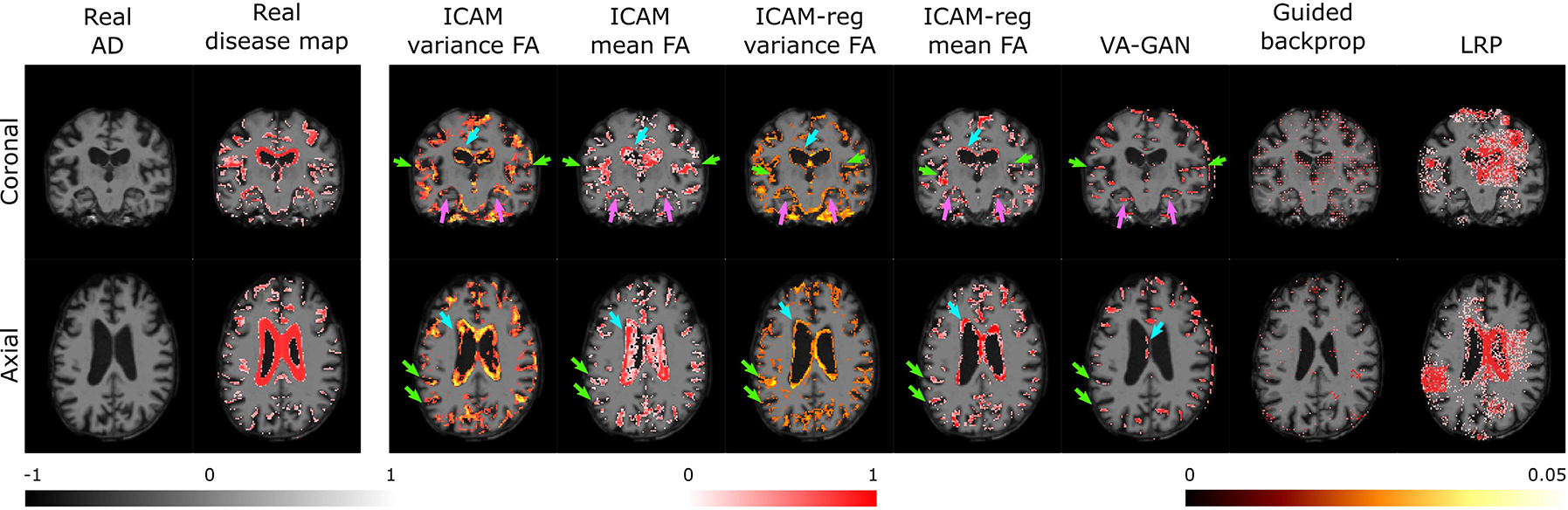
ADNI comparisons of Feature Attribution (FA) maps for different post-hoc and generative models. Results are visualised for one individual, scanned twice longitudinally, during which time the subject was known to convert. Here, the ‘Real’ (ground-truth) disease map was calculated by subtracting the difference between the two scans. ICAM (mean and variance maps) show good detection of regions known to be implicated in Alzheimer’s disease: the ventricles (blue arrows), cortex (green arrows), and hippocampus (pink arrows); results align much more closely with the ground truth than competing baseline methods.

To address these problems in [48] we developed ICAM (Interpretable Classification via disentangled representations and feature Attribution Mapping); this improved on the state-of-the-art image-to-image translation methods (Table II) [27], [30], [31], [33], [40] by disentangling class-relevant *attributes* (attr) from class-irrelevant *content* features. Sharp reconstructions were then learnt through use of a Variational Autoencoder (VAE) with a discriminator loss on the decoder (Generative Adversarial Network, GAN). This not only allows classification and generation of an attribution map from the latent space, but also a more interpretable latent space that can visualise differences between and within classes. By sampling the latent space at test time to generate an FA map, we demonstrated its ability to detect meaningful brain variation pertaining to Alzheimer’s disease (Fig. 1).

While in the past translation methods have been implemented solely for classification, regression tasks are common in medical imaging, as most diseases lie on a continuous spectrum. The key contributions of this paper are therefore as follows:

1. We extend ICAM [48] with an additional regression module to support interpretation of heterogeneous continuous phenotypes.
2. Performance is validated across three different tasks: regression of healthy ageing in the UK Biobank, neurodevelopment in the developing Human Connectome Project (dHCP), and MMSE scores from ADNI.
3. We demonstrate that adding a regression model improves the interpretability of the attribute latent space, and show that in this way ICAM-reg can provide explanations for subjects predicted as outliers by interpolating between the attribute latent space encoding of two subjects within and between age groups.
4. We perform additional experiments to validate translation, using an independent classification network, trained on real images, to verify whether the model plausibly changes the image class.

## II. Related Works

**Fig. 2.**
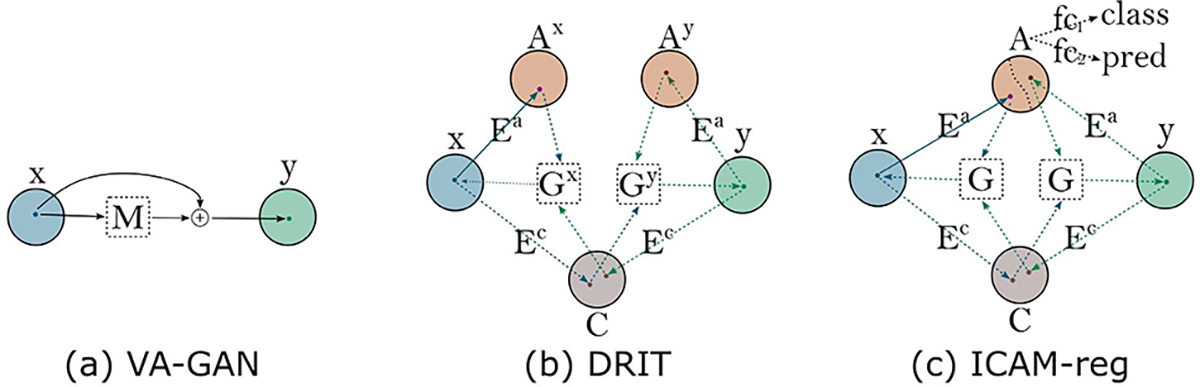
Comparison of domain mapping methods. (a) VA-GAN translates images of domain x to y. (b) DRIT can translate between domains x and y through a shared content space C, and separate attribute spaces $A^{X}$ and $A^{y}$. (c) ICAM-reg uses shared content C and attribute A spaces to translate between domains, which allows classification $f_{C_{1}}$ and regression $f_{C_{2}}$ layers to be applied to the attribute space A.

Over recent years, several deep generative approaches to image-to-image translation have emerged [41], [42], [45], [49], [50], [51], where these have been applied to many different domains, including medical imaging [40], [52], [53], [54]. Of these, Lee et al. [42], in particular, developed a domain translation network called DRIT (Fig. 2b), which constrains translation only to features specific to a class, by encoding separate class-relevant (attribute) and class-irrelevant (content) latent spaces, and employing a discriminator.

Separately, Baumgartner et al. [40] developed a conditional ‘visual attribution’ GAN which translated 3D MRI brain scans, classified with Alzheimer’s disease (AD), towards the appearance of scans with mild cognitive impairment (MCI): an intermediate state between healthy cognition and AD (Fig. 2a). This generates sharp reconstructions and realistic disease maps that overlap with ground truth patterns of longitudinal atrophy. However, the approach requires image class labels to be known *a priori* and, in the absence of a latent space, it can only produce a single deterministic output for each image, which limits the modelling of more heterogeneous features.

Accordingly, in our work ICAM [48], we extended upon the intuitions of these models to create one framework which allows simultaneous classification and feature attribution, using a more interpretable model. Compared to VA-GAN and DRIT++ [40], [42], ICAM uses 2 shared disentangled latent spaces, attribute and content, which encode for class-relevant and class-irrelevant information, respectively. The use of a shared attribute (class) latent space allows the addition of a classification layer (and in this work, also a regression layer) to the network (Fig. 2c), which enables the network to do classification and visualisation of differences between and within classes.

Other components of ICAM such as a FA map loss, L2 reconstruction loss, and a 3D attribute latent space also improve performance compared to VA-GAN and DRIT++ (as illustrated using ablation studies in [48]).

## III. Methods

**Fig. 3.**
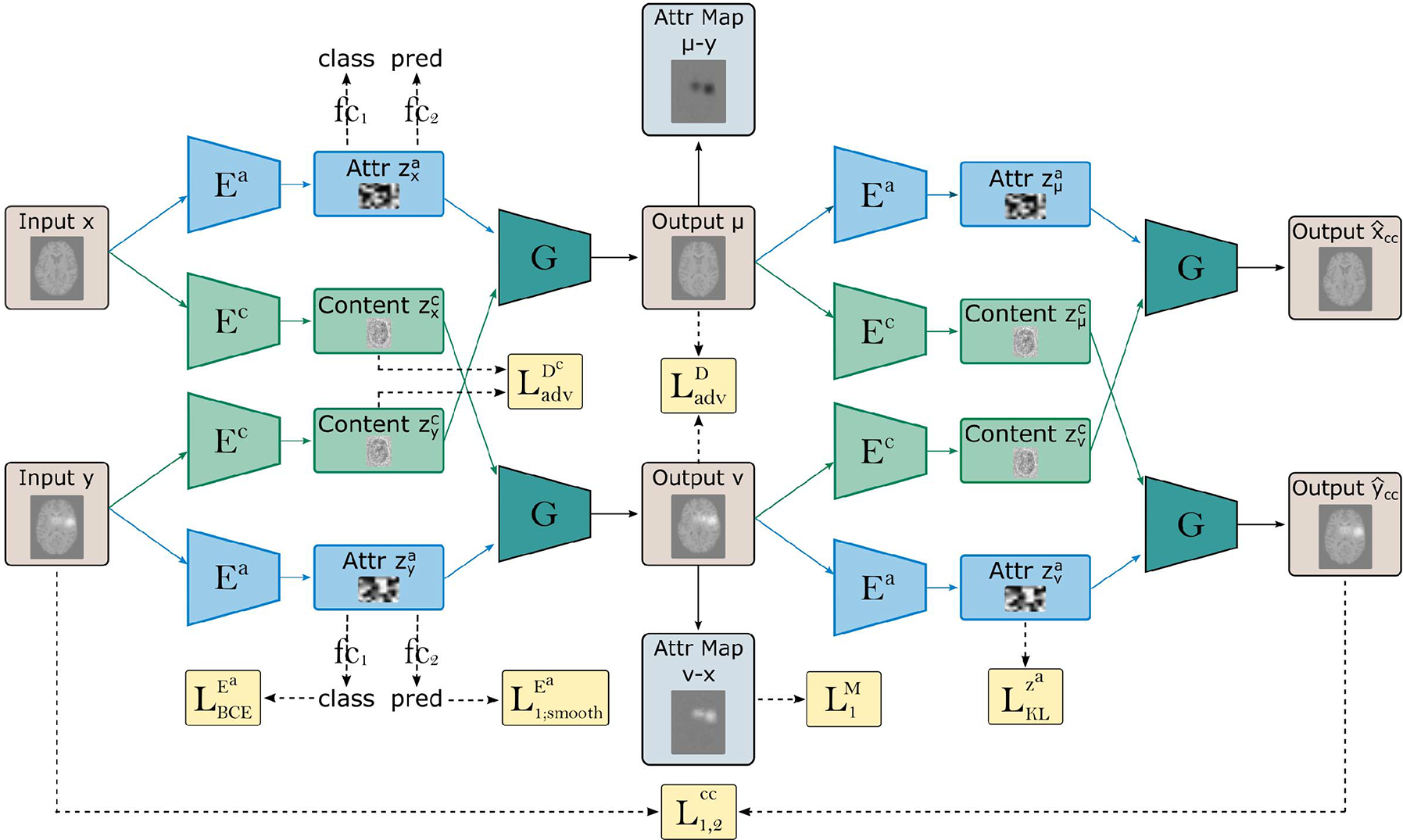
Overview of method. An example of how ICAM performs classification/regression with FA map generation for 2 given input images x (of class 0 [brain slice without lesions] and y (class 1 [brain slice with simulated lesions]). Note that $L_{adv}^{D}$ is applied to both real and generated images, and that not all losses are plotted (see Equation 5 for full objective).

The goal of ICAM [48] is to perform classification with simultaneous feature attribution, by training a VAE-GAN to swap the classes of input images (*x*, *y*) by changing only the features which are specific to the target phenotype. In this paper, we extend the method with a regression module (‘pred’ - Fig 3) to support prediction of continuous phenotypes.

### A. Content and Attribute Latent Spaces

In ICAM, domain disentanglement is achieved through encoding two separate latent spaces: a **content encoder** $\left\{E^{c}\right\}$ (latent space $z^{c}$), whose objective is to encode class-irrelevant (e.g. brain shape) information, and an **attribute encoder** $\left\{E^{a}\right\}$ (latent space $z^{a}$), whose objective is to encode all class-relevant features of disease. In both cases, the latent spaces are shared between classes or domains (i.e. $\left\{E_{c}\right.\;:\;\left.x\to C\},\;\left\{E_{c}\;:y\to C\right\}\right)$). Note, in what follows, we refer to domain or class interchangeably, in which the same meaning is implied.

For the **content encoder** $\left\{E^{c}\right\}$, class information is driven out from the latent space {C} through training of a discriminator, $\left\{D^{c}\right\}$, with **class adversarial content loss**:

(1) $$L_{adv}^{D^{c}}=E_{z_{x}^{c}}\left[log\;D^{c}\left(E^{c}(x)\right)+log\left(1-D^{c}\left(E^{c}(x)\right)\right)\right]\;\;+E_{zy}\left[log\;D^{c}\left(E^{c}(y)\right)+log\left(1-D^{c}\left(E^{c}(y)\right)\right)\right].$$

The goal of the content encoder $\left\{E_{c}\right\}$ is therefore to learn a representation whose domain cannot be distinguished by this discriminator (an approach first proposed by Lee et al., [42]). Training is also supported through L2 regularisation, to prevent explosion of gradients, and Gaussian noise (added to the last layer of the encoder) to prevent the latent space vanishing. Without this the content space goes to zero since it is the easiest way to make the space class invariant.

For the **attribute encoder** $\left\{E^{a}\right\}$, class information is driven *into* the latent space, by appending a fully connected classification layer $\left(f_{C_{1}}\right)$ with binary cross entropy loss $L_{BCE}^{E^{a}}$. In extension from our previous work [48], a **regression module** $f_{C_{2}}$) is also added, using another fully connected layer, trained using a smooth L1 loss $\left(L_{1;\mathrm{smooth}}^{E^{a}}\right)$. Importantly, when training regression modules, a complementary binary classification task must be run, in order to support the adversarial training of the generator (Sec. III-B).

The training of the attribute latent space is performed using variational inference, through application of a Kullback Leibler (KL) loss $L_{KL}^{z^{a}}$. This places a Gaussian prior over the latent variables ensuring that the attribute latent space can be sampled, which allows translation of a single subject at test time, and the generation of mean and variance maps via the use of rejection sampling (see below). During training, the prediction modules $f_{C_{1}}$ and $f_{C_{2}}$ therefore work to encourage separation of the domains within this latent space {A}, to support meaningful image translation. Further, a **latent regression loss** [42] is implemented through sampling a random attribute latent vector $\left(z_{r}^{a}\right)$ from a Gaussian distribution, then reconstructing:

(2) $$L_{1}^{z^{a}}={∥E^{a}\left(G\left(E^{c}(x),z_{r}^{a}\right)\right)-z_{r}^{a}∥}_{1}.$$

The purpose of this loss, first proposed in DRIT++ [42], is to encourage an invertible mapping between the attribute latent space and the generated outputs.

### B. Generation and Feature Attribution

**Fig. 4.**
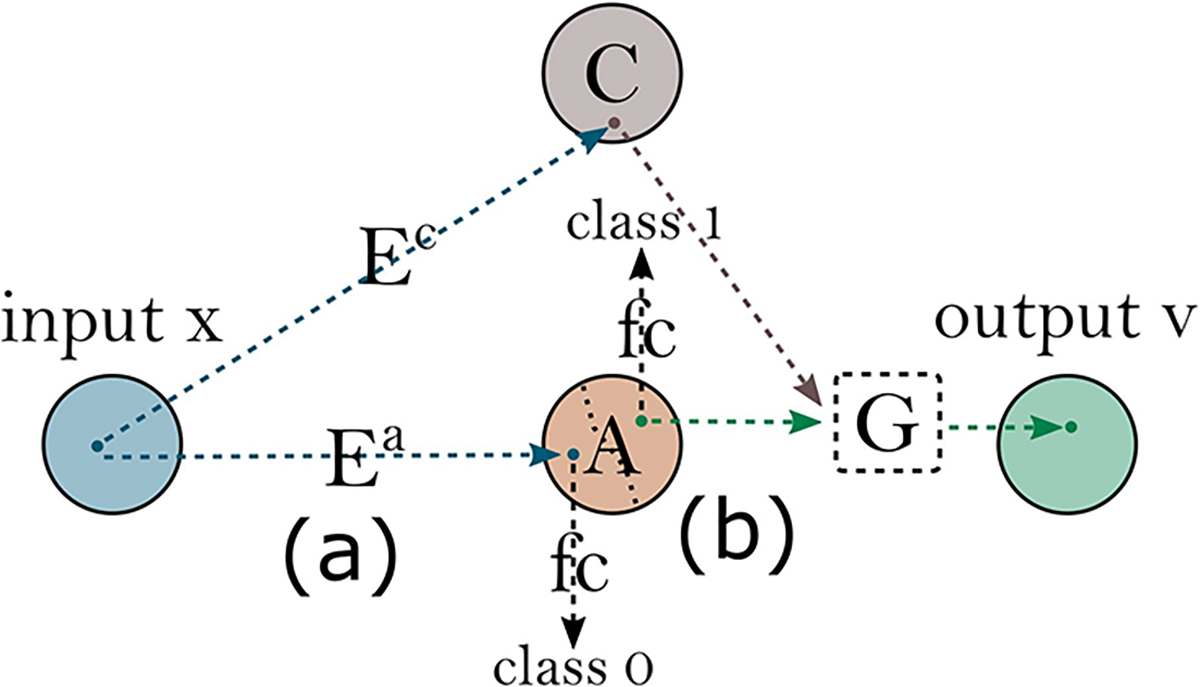
Rejection sampling during training/ testing (a) An input image is encoded into content and attribute spaces, and is passed through the classifier to identify its class (0 in this example). (b) Attribute space A is then randomly sampled until the classifier detects random vector of the opposite class. The newly sampled vector is passed to the generator along with the encoded content space to achieve translation between class 0 and 1.

Image translation and generation of FA maps is supported through the training a **generator** {G}, which learns to synthesise images conditioned on both the content and attribute latent spaces $\left(G\;:\left\{z_{x}^{c},z_{x}^{a}\right\}\to \overset{ˆ}{x}\right),\;\left(G\;:\left\{z_{y}^{c},z_{y}^{a}\right\}\to \overset{ˆ}{y}\right)$, as well as to translate between these domains. It achieves this by swapping the content latent space: $\left(G\;:\left\{z_{y}^{c},z_{x}^{a}\right\}\to \mu \right),\left(G\;:\left\{z_{x}^{c},z_{y}^{a}\right\}\to \right.v)$, which is made possible since this space is class invariant. Training of the generator is supported by optimisation of a **domain discriminator** {D} with two losses: a) a domain adversarial loss, $L_{adv}^{D}$ which seeks realistic image generation by minimising the differences between translated (fake) and real images; and b) a binary cross entropy classification loss, $L_{BCE}^{D}$, which seeks optimal classification of the two domains following translation. Disentanglement is further encouraged through **rejection sampling** of the attribute latent space during training. This checks the class of each vector randomly sampled from the attribute space (Fig. 4) to ensure that the domain discriminator is passed a simulated image of the opposing class. This is important since the objective of ICAMs adversarial training is to encourage plausible *translation* of the images.

To visualise differences between the translated images {v,μ} and the original images {x,y}, we use a **feature attribution map** {M}. This aims to retain only class-related differences between two images (or two locations in the attribute latent space) by subtracting the content from the translated output $\left(\left\{M_{x}=v-x\right\},\left\{M_{y}=\mu -y\right\}\right)$. Generation is regularised through an L1 loss $\left(L_{1}^{M}=∥M(\;){∥}_{1},\right)$ which encourages {M} to reflect a small feasible map, which leads to a realistic translated image. At test time, we generate both a mean and variance through repeated rejection sampling.

Finally, to further facilitate image generation, we apply element-wise L1 and L2 loss to the reconstructed images $\left\{\overset{ˆ}{x},\overset{ˆ}{y}\right\}\left(L_{1,2}^{rec}\right)$, and the cyclically reconstructed images $\left\{\overset{ˆ}{x_{cc}},\overset{ˆ}{y_{cc}}\right\}\;\left(L_{1,2}^{cc}\right)$. The cycle consistency term also allows training with unpaired images.

(3) $$L_{1}^{rec}+L_{2}^{rec}=E_{x,y}\left[{\left\|G\left(E^{c}(x),E^{a}(x)\right)-x\right\|}_{1}\right.\;\;\;\;\;\;\;\;\;\;\;\;\;\;\;\;\;\;\;\;\;\;\;\;\;\;\;\;\;\;\;\;\;\;\;\;\;\;\left.+{\left\|G\left(E^{c}(y),E^{a}(y)\right)-y\right\|}_{1}\right]\;\;\;\;\;\;\;\;\;\;\;\;\;\;\;\;\;\;\;\;+E_{x,y}\left[{\left\|G\left(E^{c}(x),E^{a}(x)\right)-x\right\|}_{2}\right.\;\;\;\;\;\;\;\;\;\;\;\;\;\;\;\;\;\;\;\;\;\;\;\;\;\;\;\;\;\;\;\left.+{\left\|G\left(E^{c}(y),E^{a}(y)\right)-y\right\|}_{2}\right],$$

(4) $$L_{1}^{cc}+L_{2}^{cc}=E_{x,y}[{∥G\left(E^{c}(v),E^{a}(\mu )\right)-x∥}_{1}\;\;\left.+{∥G\left(E^{c}(\mu ),E^{a}(v)\right)-y∥}_{1}\right]\;\;\;+E_{x,y}\left[{∥G\left(E^{c}(v),E^{a}(\mu )\right)-x∥}_{2}\right.\;\;\left.+{∥G\left(E^{c}(\mu ),E^{a}(v)\right)-y∥}_{2}\right].$$

This means the **full objective function** of our network is:

(5) $$\underset{G,E^{c},E^{a}}{\min}\underset{{}_{D,D^{c}}}{\max}\;\lambda_{D^{c}}L_{adv}^{D^{c}}+\lambda_{D}L_{adv}^{D}+\lambda_{D_{BCE}}L_{BCE}^{D}\;\;\;\;\;\;\;\;\;\;\;+\lambda_{BCE}(L_{BCE}^{E^{a}}+L_{1;smooth}^{E^{a}})+\lambda_{KL}L_{KL}^{z^{a}}+\lambda_{M}L_{1}^{M}\;\;\;\;\;\;\;\;\;\;+\lambda_{z^{a}}L_{1}^{z^{a}}+\lambda_{\mathrm{rec}}\left(L_{1}^{rec}+L_{1}^{cc}+L_{2}^{rec}+L_{2}^{cc}\right).$$

### C. Training Details

ICAM is trained in a similar fashion to Lee et al. [42]. For each iteration, the content discriminator is updated twice, followed by the update of the encoders, generators, and domain discriminators (i.e. each training iteration uses 3 batches to perform these updates). For each update of the generator, one input is selected for each class (e.g. 2 inputs including class 0 and 1). All experiments use the following hyperparameters: learning rate for content discriminator = 0.00004, learning rate for the rest = 0.0001, Adam optimiser with betas = (0.5, 0.999), $\lambda_{D^{c}}=1,\;\lambda_{D}=1,\;\lambda_{BCE}=10,\;\lambda_{KL}=0.01,\;\lambda_{M}=10,\;\lambda_{z^{a}}=1,\;\lambda_{\mathrm{rec}}=100,\;\lambda_{D_{BCE}}=1$, for discriminator optimisation, and $\lambda_{D_{BCE}}=5$ for generator optimisation. These parameters were optimised for a 2D data set of simulated cortical lesions, as previously described in [48]. Regression experiments use a network pre-trained for classification, refined with addition of the regression loss.

## IV. Results

We evaluate the performance of ICAM-reg through three experiments: 1) brain age prediction (using data from UK Biobank); 2) regression of birth age (using neonatal data from the developing Human Connectome Project - dHCP); and 3) prediction of MMSE scores (using data from ADNI). We compare against VA-GAN (for ADNI and UK Biobank), and against post-hoc saliency methods (for ADNI). All experiments were trained with PyTorch [55] using NVIDIA TITAN GPUs. For an extensive ablation study and evaluation of the impact of changing ICAM hyperparameters please refer to [48] and the project GitHub page.^1^

### A. Brain Age Prediction for the UK Biobank Cohort

#### 1) UK Biobank Dataset and Training:

The performance of ICAM and VA-GAN for brain age prediction was validated using T1 MRI data from healthy subjects (aged 45–80 years) acquired for the UK Biobank [56], [57]. T1 image processing (see also [56]) involved bias correction using FAST [58], brain extraction using BET [59] and linear registration to MNI space, using FLIRT [60]. The input into the networks was resized to 128 × 160 × 128 voxels, and normalised in range [0, 1]. For our classification experiments we used 11,735 MRI volumes, with a ‘young’ class defined as 45–60 years (average age 54.6±3.4 years) and an ‘old’ class defined as 70–80 years (average age 73.0±2.2 years). Young subjects were separated into training, validation, and testing set sizes of: 6706, 373 and 372. Older subjects were separated into training, validation, and testing set sizes of 3856, 214 and 214.

For regression we used all available subjects (21,388), where adversarial training of the classifier was supported by defining two classes at the mid-range (45–65 and 65–80); subjects corresponding to the young class (average age 57.6±4.8 years) were separated into training, validation, and testing sets with sizes: 10715, 595 and 595; subjects corresponding to the old class (average age of 70.0±3.3 years) were separated into training, validation, and testing sets with sizes: 8535, 474 and 474. Performance on FA map generation was compared against VA-GAN trained using the default parameters provided in [40]. Both networks were trained for 50 epochs.

#### 2) UK Biobank Results:

In previous work [48], we compared feature attribution with ICAM and VA-GAN, and found that ICAM generated FA maps that better matched patterns of ‘ground-truth’ atrophy observed between longitudinally acquired scans (see also Fig 1). In this work, to demonstrate more conclusively whether translation by ICAM and VA-GAN fully changes the image class, we trained an independent binary age classifier (old vs young) using the same architecture as the ICAM attribute encoder. The classifier was trained using the ‘Real’ 3D T1 MRI images (Table I, row 1), or on outputs generated by ICAM (Table I, row 2) and VA-GAN (Table I, row 3), with training and test sets kept as before. Results (Table I) show that classification with images generated by ICAM performs slightly worse than the real data (82.2% compared to 93.8%), which is to be expected in a complex 3D generation task. By contrast, VA-GAN outputs perform much worse (12.2%). Note that because VA-GAN can only translate in one direction, it has only 1 result in the table.

**Fig. 5.**
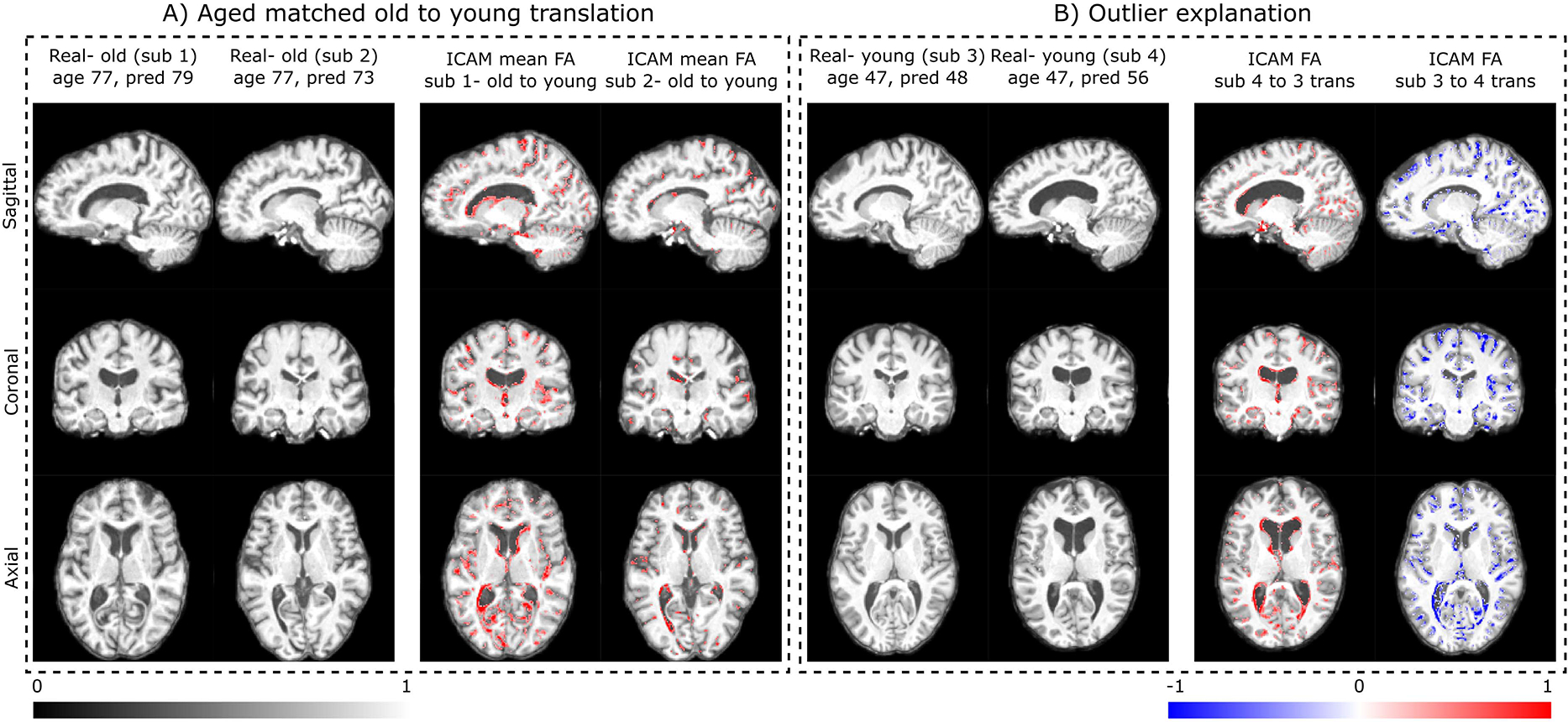
UK Biobank regression: here we show two different ways in which FA maps derived from ICAM-reg can be used to explain outlier predictions. In the left box we show, FA maps resulting from translating two individuals (true age 77) towards a classification of young (using rejection sampling). In this example subject 1 is predicted as older (79) and subject 2 is predicted as younger (73); this correlates with the FA maps, which show greater age-related changes for subject 1. On the right we show FA maps derived from interpolating between two subjects within the attribute latent space. Again both have the same true age but subject 4 is predicted as much older than subject 3. The FA maps provide an explanation for this difference, showing that to translate subject 4 towards subject 3 it is necessary to fill in the ventricles and reduce cortical atrophy - all changes associated with healthy ageing.

Next, we trained ICAM-reg’s regression layer to predict ages of the MRI brain scans: resulting in a precision of of 2.20 ± 1.86 mean absolute error (MAE) (Fig. 7). We found that the resulting FA maps explained outlier predictions well. For example in Fig. 5 A), FA maps of two subjects, scanned at 77 years, and translated to resemble the younger age class, indicate greater age-related changes (e.g. ventricular and cortical atrophy) in subject 1 (which is predicted as older - 79) relative to subject 2 (which is predicted as younger - 73). In B) 2 subjects from the young group are directly compared by translating between them. In this case, subject 4 is predicted to be much older than their true age (predicted=56; true=47 years); whereas, subject 3 has predicted age 49, close to their true age (47). Evidence for the outlier prediction of subject 4 is presented through the translation, indicating the presence of larger ventricles, hippocampal atrophy and cortical shrinking (relative to the more typical presentation of subject 3).

**Fig. 6.**
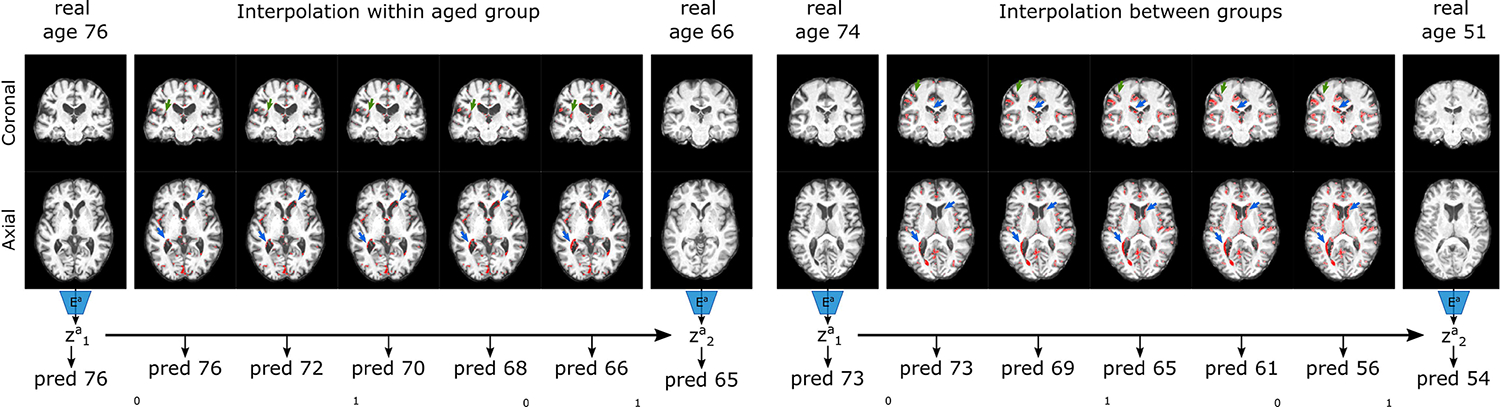
Biobank interpolation between and within groups. Here, we show an example of interpolation of the attribute latent space, with the corresponding FA maps for each vector. We overlay the interpolated FA maps on the original image, with red maps indicating an increase pixel intensity. We first encode each image to its attribute latent space (using $E^{a}$), and get an age prediction. We then linearly interpolate between these two spaces, and get an age prediction and FA map for each vector. We demonstrate that our ICAM-reg model can successfully achieve interpolation between and within groups (i.e. within the aged group, and between the aged and young groups). We find that we get both smoothly interpolated FA maps, and interpolated age predictions between two subjects. The green arrows point to the cortex, and blue arrows point to the ventricles.

In addition, we investigated the improvement in separation of the model’s latent space afforded through regression (Fig. 8), where this result is further underlined in Fig. 6, which shows clearly that interpolation between images of two different ages smoothly translates both predicted ages and FA maps, for the generated images.

Finally, since it is required by the ICAM-reg framework to train regression tasks with complementary binary classification, we investigated whether imbalancing the classification (by moving the cut-off between classes) would impact the performance and interpretability of the FA maps. We ran a smaller version of the network (output channel dimensions 13:26:52 instead of 16:32:64), for three different thresholds: one at 60 years (where the young age group is 40–60 and the old age group is 60–90); one at 65 years (where the young age group is 40–65 and the old age group is 65–90); and one at 70 years (where the young age group is 40–70 and the old age group is 70–90). Since we now had different training, validation and testing splits, we selected a subset of 100 test examples which overlapped across all experiments. Results in Fig. 9 show ICAM-reg FA maps generated for one randomly selected subject of age 66. These return very similar FA mean maps for each experiment, despite the subject belonging to a different age classification each time. Importantly, we observe similar changes to key areas associated with healthy ageing. We did find that age prediction error varied across experiments: 3.27 (threshold 60), 3.67 (threshold 65) and 3.77 MAE (threshold 70); this may reflect the use of different training splits. We therefore conclude that while threshold selection is unlikely to lead to large differences in prediction and FA map generation, it could be a hyperparameter that can be tuned.

### B. dHCP Experiments

#### 1) dHCP Dataset and Training:

In this experiment we sought to demonstrate that ICAM-reg can work well for prediction of challenging phenotypes, and detection of focal lesions, from relatively small, heterogenous, datasets. We used 699 3D T2 MRI scans from the dHCP [61], [62]: an open data set of multimodal brain scans acquired from preterm and term neonates. Here, preterm is defined as birth prior to 37 weeks gestational age (GA), where some preterm neonates were scanned twice: at birth and at term equivalent age. The data set includes 143 preterm images (class 1, mean gestation age at birth: 31.8 ± 3.85 weeks, mean post-menstrual age at scan: 41.0 ± 1.99 weeks) and 556 term controls (class 0, mean age at birth: 40.0 ± 1.27 week, mean post-menstrual age at scan: 41.4 ± 1.74). In this experiment ICAM-reg was trained to classify between preterms and terms, and predict birth age from the term age scan (i.e. scans acquired after 37 weeks post-menstrual). Examples were split into train, validation and test sets according to a 446:55:55 split (for term subjects) and 115:14:14 split (for preterm subjects).

Image pre-processing involved using diffeomorphic multimodal (T1w/T2w) registration (ANTs SyN) to estimate nonlinear transforms to a 40 week template from the extended atlas [62], [63], [64]. This was necessary to allow the network to train, since without this step the network was challenged by stark changes in image appearance across the cohort, caused by rapid tissue maturation, and further confounded by the relatively small and imbalanced nature of the data set. For related reasons (to preserves age-related tissue maturational differences), images were rescaled to [0,1] by normalising across the intensity range of the entire group. Images were then brain extracted (using blurred masks), and CSF, ventricles and the skull were removed in order to focus the attention of the model on brain tissue differences between the groups.

ICAM-reg was pre-trained on UK Biobank data; then trained on dHCP birth age regression for a further 1000 epochs, using the same hyperparameters. Performance was compared against a baseline CNN network, trained with same architecture as $E^{a}$, using smooth L1 loss, with Adam optimiser (learning rate = 0.0001, betas = [0.5, 0.999]) for 1000 epochs.

#### 2) dHCP Results:

**Fig. 7.**
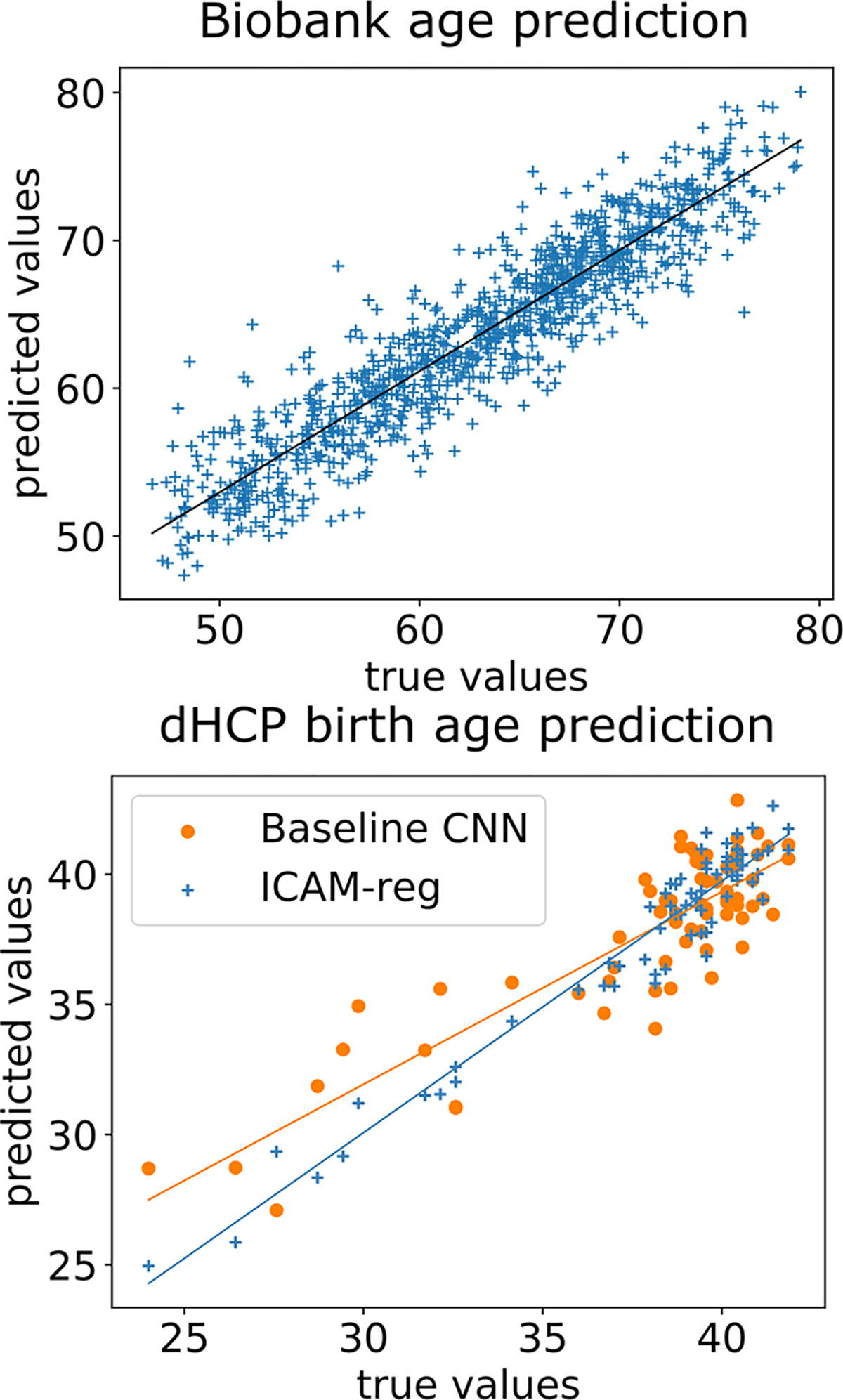
Biobank (top) and dHCP (bottom) age prediction on the test dataset using ICAM-reg. For biobank, the age prediction error is 2.20 ± 1.86 MAE. For dHCP, the birth age prediction MAE is 0.806 ± 0.634, for ICAM-reg, and 1.525 ± 1.160, for the baseline network.

Results are shown in Figs. 7 and 10. We report a birth age prediction MAE of 0.806 ± 0.634 for ICAM-reg vs 1.525 ± 1.160 for the baseline CNN (Fig. 7). In addition, we report a higher correlation coefficient for ICAM-reg (Spearman correlation test, *p* < 0.0001, 0.873 for ICAM-reg and 0.695 for the baseline network).

For qualitative analysis we tested ICAM-reg on previously unseen images of subjects with punctate white matter lesions (PWML), which are commonly seen in preterm babies [20], [65], to test whether these would be detected in the FA maps. The results are shown in Fig.10 with yellow arrows pointing at the lesions. Quantitative analysis of the detection rates for these lesions resulted in a recall of 0.805 ± 0.078 and precision of 0.004 ± 0.004, for term subjects, and recall 0.734±0.102 and precision 0.004±0.005 for preterm subjects. Note, unlike for the ADNI and biobank results, here the FA maps were thresholded at 0.01 to remove some of the image generation noise from the calculation, and binarise the masks. Binarisation was necessary as we sought to test purely whether lesions were being detected (or not) through calculation of precision and recall scores. We tested several thresholds (range 0–0.25) and reported results with the most optimal threshold for recall-precision trade-off. These results suggest ICAM-reg consistently detects lesions in both cohorts.

### C. ADNI Experiments: Ground-Truth Evaluation of FA Maps

In the final experiment, we demonstrate the performance of ICAM’s feature attribution against ground truth maps of disease progression estimated for AD to MCI conversion using the ADNI dataset, and extend [48] to explore modelling regression of MMSE scores. The MMSE is a test that is commonly used for the assessment of dementia by examining memory, thinking and problem-solving abilities of a patient. The score ranges between 1–30 with scores of 25–30 considered normal, 21–24 indicates mild dementia, 10–20 indicates moderate dementia, and 9 or lower indicates severe dementia.

#### 1) ADNI Dataset:

The data used in this study was obtained from the Alzheimer’s Disease Neuroimaging Initiative (ADNI) database (adni.loni.usc.edu), first launched in 2003, and led by Principal Investigator Michael W. Weiner, MD [66]. We used 1,053 3T T1 images, pre-processed with N4 bias correction [67], brain extracted using Freesurfer [68] and rigidly registered to the MNI space using Niftyreg [69]. Images were normalised in range [−1, 1], and resized to 128 × 160 × 128 voxels.

For our classification experiments (used for comparisons in Table II) we split the dataset into AD and MCI classes, with 257 AD and 674 MCI volumes used for training. For our regression experiments we split the dataset into AD and MCI classes, with 223 AD and 626 MCI volumes used for training. The average age of training subjects was 74.91±8.1 (for AD) and 71.97 ± 7.8 (for MCI), with average MMSE scores of 23.02 ± 2.6 (AD) and 27.75 ± 2.6 (MCI). For testing and validation, the same 122 subjects (61 each) were used for both classification and regression experiments. The average age of validation subjects was 75.88±6.8 (for AD) and 73.67±7.0 (for MCI) with mean time between scans of 2.20±0.9 years. The average validation MMSE scores were 23.72 ± 4.3 (AD) and 26.95 ± 2.8 (MCI). The average age of test subjects was 75.63 ± 7.6 (for AD) and 73.44 ± 7.6 (for MCI) with mean time between scans of 2.19 ± 1.0. The average test MMSE scores were 24.21 ± 4.1 (AD) and 26.77 ± 3.0 (MCI).

#### 2) ADNI Training:

ICAM-reg experiments were performed to jointly classify AD from MCI whilst also regressing MMSE, where it is assumed that these two tasks are correlated. We compare performance against ICAM [48], trained purely on MCI-AD classification, and a range of baseline methods: VA-GAN [40], Grad-CAM, guided Grad-CAM [27], guided backprop [31], integrated gradients [30], occlusion [33] and Layer-wise Relevance Propagation (LRP) [32].

VA-GAN was trained using default parameters and post-hoc methods were applied following training of a simple 3D ResNet with 4 down ResNet blocks, and a fully connected layer for classification of AD vs MCI. Saliency maps were then generated using the captum library [70], where: Grad-CAM was implemented on the last convolutional block of the ResNet (with a size of 4 × 5 × 4) and was up-sampled to the input size for visualization; integrated gradients was implemented by considering a baseline volume with constant value of 0, and the integral was computed using 200 steps; and occlusion was implemented using occlusion blocks with value 0, size 10 × 10 × 10 and stride 5.

All networks (including VA-GAN) were trained for 300 epochs. Both ICAM networks were then further refined, for another 200 epochs, using updated lambdas ($\lambda_{\mathrm{rec}}=10$, and $\lambda_{BCE}=20$). It was not possible to refine VA-GAN any further because generator and discriminator losses went to zero during training (often after 150 epochs). The baseline classifier network was trained for 50 epochs with learning rate of 0.0001, SGD with momentum of 0.9, for 50 epochs, and using a weighted BCE loss (to account for class-unbalanced training).

Methods were evaluated by comparing the overlap of the proposed FA maps against ground truth, obtained by subtracting the difference between test scans (acquired before and after conversion) following rigid alignment. All ground truth maps and FA maps were masked to ensure that the returned normalised cross correlation (NCC) values reference brain tissue only. We also report the classification and regression performance of the ICAM-reg and ResNet models only (since VA-GAN does not support supervised learning).

#### 3) ADNI Results:

Results comparing the NCC of the proposed FA maps with ‘ground-truth’ disease maps (Table II) show that all versions of ICAM outperform VA-GAN, and post-hoc saliency methods. We further demonstrate qualitatively in Fig. 1, that relevant areas of brain atrophy are detected using ICAM by comparing with disease map (ground truth). Classification accuracy of ICAM-reg for the AD vs MCI prediction was 60.7%; whereas for the simple ResNet prediction was 61.7%. At the same time, regression of the MMSE score returns MMSE prediction of 2.82±2.14 mean absolute error. Importantly, we cannot compare this to VA-GAN or other FA methods, as they cannot be normally applied to regression tasks.

## V. Discussion

In our previous work [48] we developed a novel framework, ICAM, for classification with feature attribution, and showed that it outperforms state-of-the-art feature attribution methods on classification tasks for individual subject feature detection. In this work, we extended ICAM to include a regression module, ICAM-reg. We then sought to test whether, when trained on a large dataset (UK Biobank), ICAM-reg could learn to disentangle its attribute latent space, so as to support meaningful interpolation between images, and generate subject-specific explanations for outlier predictions. We also demonstrated that ICAM-reg can work on much smaller and more heterogeneous datasets (dHCP, ADNI), while continuing to detect relevant features not explicitly defined during training, i.e. white matter lesions in the dHCP neonatal data; and predict clinically relevant phenotypes such as age at birth (dHCP) and cognitive test scores (ADNI).

It is important to stress that for all examples ICAM-reg runs simultaneous regression *and* classification, where the choice of classifier must be complementary. This is because the backbone of the algorithm remains an image-to-image translation network that trains discriminator networks to change the class of input images. To this end, we tested the impact of selecting different thresholds for converting the regression task to a classification task (i.e. splitting the dataset into 2 groups) using the UK Biobank dataset, and have found that threshold selection can have a small impact on performance and the interpretability of the FA maps, and thus can be considered a hyperparameter that can be tuned.

Nevertheless, through experiments on UK Biobank, we demonstrate that ICAM can more comprehensively translate the class of input images, relative to VA-GAN [40] (Table I). Further visual comparison (Fig 1) shows that while VA-GAN is only able to slightly modify the images by changing pixel intensities in order to generate FA maps, ICAM can drastically change the input image in order to change its class, and thus also generate more reliable FA maps. Fig. 1 shows that qualitatively the pattern of atrophy detected by ICAM aligns with the ‘Real’ (ground truth) disease map, and with known patterns of brain tissue loss reported for AD, which starts in the hippocampus, and progresses from medial to lateral temporal lobes, to the parietal and frontal lobes in late stages [71]. A common and easily observed side-effect of tissue loss is the growth of fluid filled spaces (such as the ventricles) which is also picked up here. While there are undoubtedly interaction effects from age-related decline, the mean time interval between scans is not long (around 2 years), and these results qualitatively agree with previous papers that have attempted to disentangle pathological atrophy from healthy ageing [71], [72], [73]. By contrast, VA-GAN was less able to detect atrophy of the more heterogeneous regions of the cortex. Equally the best performing post-hoc saliency methods (guided-backprop and LRP) also show less sensitivity, with LRP also returning asymmetric predictions, which may reflect the inability of post-hoc methods to capture redundant features.

**Fig. 8.**
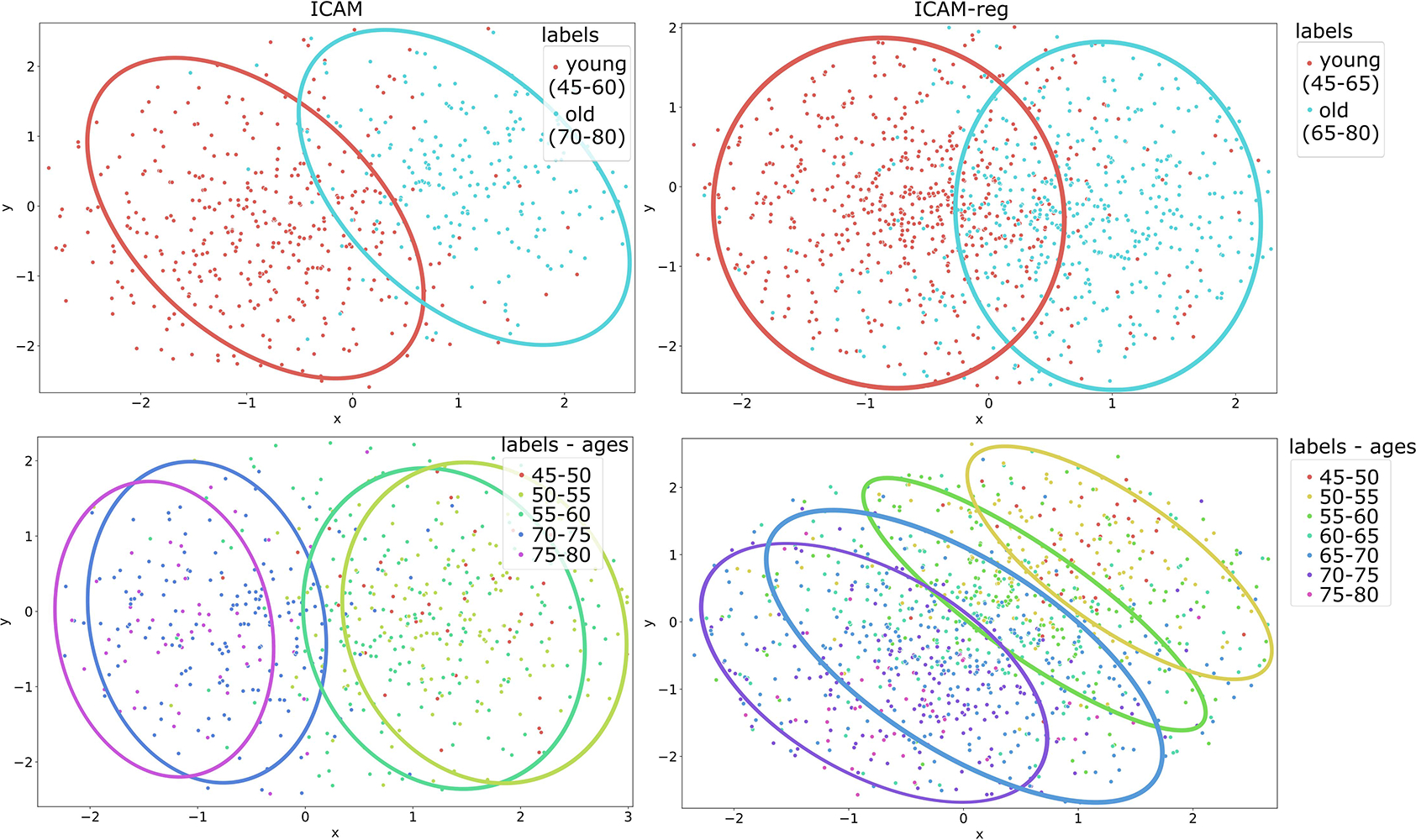
tSNE plots comparing the latent space of ICAM (left) and ICAM-reg (right). Top row shows the separation of old and young classes. Bottom row shows the distribution binned for every 5 years. In each case the results are plotted for the test subjects of each model.

**Fig. 9.**
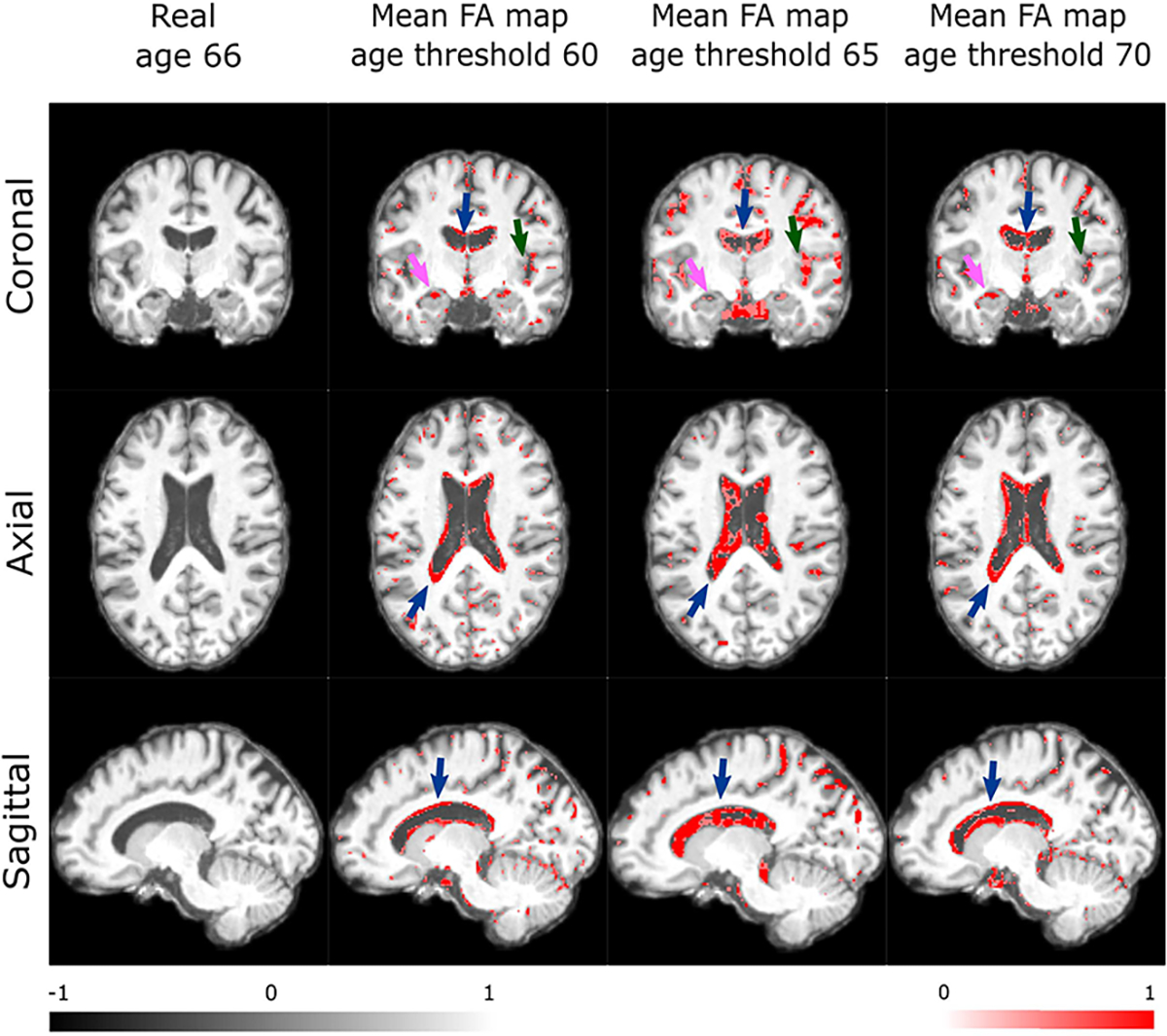
FA map robustness: ICAM-reg was run three times, with three different thresholds determining the classification splits. Columns 2–4 show FA mean maps for each experiment. Maps are shown for the same subject (aged 66 years) translated from old to young. Features stay broadly consistent with similar changes to key areas associated with healthy ageing: ventricles (blue arrows), hippocampus (pink arrows), and cortex (green arrows).

In separate experiments, we show that brain age prediction by ICAM-reg (2.20 ± 1.86 MAE, Fig. 7) performs highly competitively relative to other deep learning methods trained on age prediction in UK Biobank, with reported test MAE scores of 2.14±0.05 [74], 2.71±2.10 (female) and 2.91±2.18 (male) [75], and 4.006 [76]. Alongside the age prediction, we find that ICAM-reg can provide meaningful and individual explanations for old and young classification, as well as outlier predictions (Figs. 5, 6). We also demonstrated that our regression model has a more interpretable latent space than our previous model [48], through use of a tSNE comparison (Fig. 8), and demonstrated interpolation of the latent space between and within groups (Fig. 6).

**Fig. 10.**
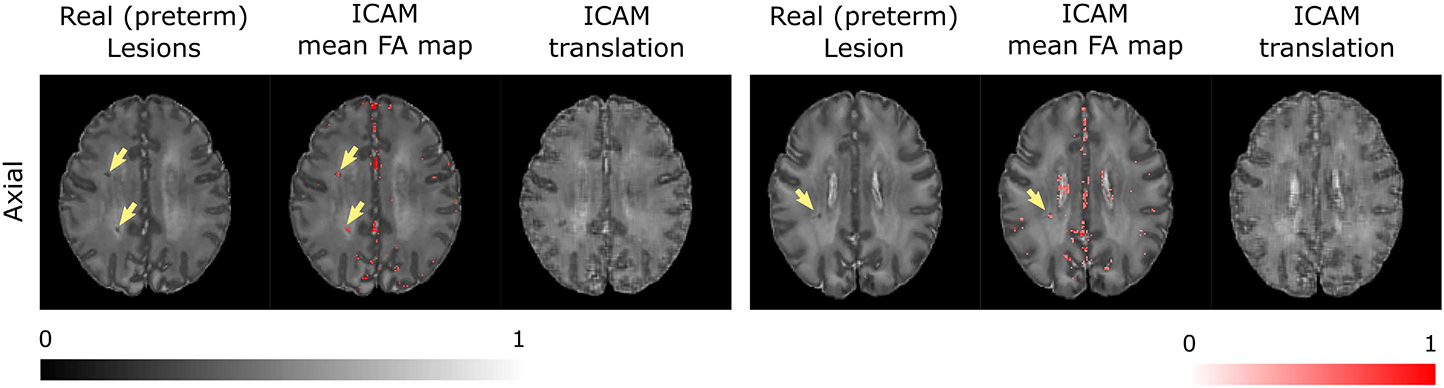
dHCP results. Here we show detection of punctate white matter lesions (yellow arrows) on previously unseen images by ICAM-reg.

In our dHCP experiments, we compared our regression model to a baseline CNN that has the same architecture as our attribute encoder and found that ICAM-reg performs better than the baseline CNN on birth age prediction (Fig. 7). Despite significant class imbalance ICAM-reg’s error (Fig. 7) is approximately consistent across the age range. This may be attributed to the fact that each forward pass through the network takes an example from each class, meaning that each class is sampled in a balanced way during training. At the same time, the model returns subject specific FA explanations of the predictions, which consistently detect punctate white matter lesions, within individuals (a known feature of preterm birth, Fig. 10). These are detected despite stark changes in image intensity and appearance over this neonatal period. This is further demonstrated by our qualitative experiments where we computed precision and recall scores between ground truth maps and generated FA maps, and found a high recall (i.e. high rate of lesion detection) and low precision (i.e. high amount of false positives). That precision is extremely low is not surprising since ICAM-reg is trained to predict birth age, therefore the FA maps should be expected to pick up on the diffuse tissue maturation changes known to exist between the two groups rather than explicitly focusing on the PWMLs.

For ADNI we show that ICAM-reg can predict cognitive scores related to Alzheimer’s (MMSE scores), and provide meaningful FA map explanations that highlight individualised patterns of brain atrophy better than baseline methods (Fig. 1). One challenge with using longitudinal brain atrophy, as ground truth for validation, is that this also incorporates age-related changes [11]. This may be why NCC scores are reduced for ICAM-reg (based on MMSE) relative to ICAM (based on disease classification only).

Moreover reported classification of AD versus MCI, for both ICAM-reg and the baseline ResNet does not achieve state-of-the art performance, which some studies report as high as 76% [77], [78]. While optimising classification and regression scores was not the main objective of this paper, the relatively strong performance on UK Biobank age regression suggests that results on ADNI might be improved if the confounding effects of age, sex and scanner site were removed by for example, adding additional deconfounding modules to the network. Improved performance may also be achieved through better balancing of MMSE values across the training and test sets [79], addressing MMSE heteroscedasticity through use of through a different loss, and inclusion of additional modalities for example T2 FLAIR or PET.

Finally, there are several challenges that could still be investigated in future work. First, while ICAM has been applied to regression and binary classification problems, it has still not been tested on multi-class datasets. Second, while ICAM shows some potential for subject specific modelling of disease progression, for example conversion of progressive MCI to full AD, or projecting the neurological impact of preterm birth, considerable more effort would be required prior to clinical translation to ensure the model is unbiased and generalises across scanners and sites. Finally, it is still challenging to apply ICAM to small and diverse data sets, particularly developmental cohorts, across which tissue intensities and brain shape change very rapidly. This was addressed for the dHCP experiments in this paper by using non-linear registration to remove gross brain shape variation and thus reduce the amount of variation the network had to learn. In future, these challenges could be addressed via application of GAN augmentation techniques [80] to increase training data for smaller datasets, and latent space clustering strategies to further encourage disentanglement of imbalanced classes [81].

## Figures and Tables

**TABLE I:** Biobank Generation Experiment Comparing Accuracy Score for Classification (Young Vs Old) of Real, ICAM Generated, and VA-GAN Generated Data. Note That Because VA-GAN Can Only Do Old to Young Translation, It Has Only 1 Result in the Table

Dataset	Accuracy - young	Accuracy - old

Real	**0.938**	0.859
ICAM (translated)	0.822	**0.865**
VA-GAN (translated)	0.122	N/A

**TABLE II:** ADNI Experiments Comparing Baselines With ICAM. Networks Are Compared Using Normalised Cross Correlation (NCC) Between the Absolute Values of the Attribution Maps and the Ground Truth Maps. The Positive NCC (+) Compares the Ground Truth Map to the FA Map When Translating Between Class 0 (MCI) to 1 (AD), and Vice Versa for the Negative NCC (−). Values Reported Are the Mean and Standard Deviation Across the Test Subjects

Network	NCC (−)	NCC (+)

Guided Grad-CAM [27]	0.244 ± 0.047	0.339 ± 0.068
Grad-CAM [27]	0.321 ± 0.059	0.461 ± 0.086
Occlusion [33]	0.360 ± 0.037	0.354 ± 0.057
Integrated gradients [30]	0.378 ± 0.064	0.404 ± 0.059
LRP [32]	0.390 ± 0.033	0.387 ± 0.039
Guided backprop [31]	0.541 ± 0.054	0.532 ± 0.052
VA-GAN [40]	0.653 ± 0.142	N/A
ICAM-reg	0.655 ± 0.086	0.611 ± 0.059
ICAM	**0.683** ± **0.097**	**0.652** ± **0.083**

## Footnotes

1
https://github.com/CherBass/ICAM#ablation-and-parameter-optimisation
